# Central Nervous System Complications in Children Receiving Chemotherapy or Hematopoietic Stem Cell Transplantation

**DOI:** 10.3389/fped.2017.00105

**Published:** 2017-05-15

**Authors:** Duccio Maria Cordelli, Riccardo Masetti, Daniele Zama, Francesco Toni, Ilaria Castelli, Emilia Ricci, Emilio Franzoni, Andrea Pession

**Affiliations:** ^1^Child Neurology and Psychiatry Unit, University of Bologna, S. Orsola-Malpighi Hospital, Bologna, Italy; ^2^Department of Pediatrics, “Lalla Seràgnoli”, Hematology-Oncology Unit, University of Bologna, Bologna, Italy; ^3^Neuroradiology Department, IRCCS Institute of Neurological Sciences, Bellaria Hospital, Bologna, Italy

**Keywords:** neurotoxicity, neuroradiologic, posterior reversible encephalopathy syndrome, thrombosis, aspergillosis, methotrexate, central nervous system toxicity, chemotherapy

## Abstract

Therapy-related neurotoxicity greatly affects possibility of survival and quality of life of pediatric patients treated for cancer. Central nervous system (CNS) involvement is heterogeneous, varying from very mild and transient symptoms to extremely severe and debilitating, or even lethal syndromes. In this review, we will discuss the broad scenario of CNS complications and toxicities occurring during the treatment of pediatric patients receiving both chemotherapies and hematopoietic stem cell transplantation. Different types of complications are reviewed ranging from therapy related to cerebrovascular with a specific focus on neuroradiologic and clinical features.

## Introduction

Therapy-related central neurotoxicity represents a vast and heterogeneous chapter. Due to the improved survival of pediatric patients with cancer, its importance is becoming more and more relevant in defining the quality of life of such patients. In fact, clinical consequences of toxicity on central nervous system (CNS) may vary from very mild and transient symptoms to extremely severe and debilitating, or even lethal syndromes. CNS complications can occur also in children affected by non-malignant disorders receiving chemotherapy in a setting of hematopoietic stem cell transplantation (HSCT), as this latter procedure has become an increasingly frequent indication for many congenital and acquired pediatric diseases. Involvement of CNS may be due to direct toxicity of specific agents or may be related to the complex interplay of multiple circumstances, as is the case of posterior reversible encephalopathy syndrome (PRES). Moreover, infections and cerebrovascular events can determine important sequelae in patients undergoing a treatment including chemotherapy and/or HSCT. Since discontinuation of the responsible agent is often necessary, prompt recognition of the etiology is fundamental. Nevertheless, this may be a hard task since neurotoxicity of antineoplastic treatments often simulates complications related to the disease itself, such as CNS recurrence or metastases. In this review, we propose a comprehensive guide for evaluating CNS toxicity of children receiving chemotherapy and/or undergoing HSCT, focusing on specific neuroradiologic features and particular clinical aspects of iatrogenic, non-iatrogenic, infective, and vascular CNS complications.

## Posterior Reversible Encephalopathy Syndrome

Posterior reversible encephalopathy syndrome is a clinical neuroradiological entity characterized by the presence of seizures, headache, vomiting, visual disturbances, and impaired consciousness, associated with MRI findings of bilateral gray and white matter edema typically prevalent in the posterior regions of the brain (1, 2). PRES is generally considered reversible; nevertheless, if not promptly diagnosed and treated, it can lead to severe complications and permanent neurological damage. In children, PRES has become increasingly observed in different clinical scenarios, such as renal, autoimmune and hematologic disorders (e.g., hemophagocytic lymphohistiocytosis) but remains mostly described as a complication of chemotherapy and allogeneic HSCT with an incidence ranging between 1 and 10% (3). PRES may occur in children during treatment for leukemia and solid tumors, but it is also described as a neurological complication in children receiving HSCT for non-malignant disorders. The pathophysiology of PRES remains uncertain and is still discussed. Two different theories have been formulated to explain the development of cerebral vasogenic edema (4). In the first theory, hypertension is the main factor: this theory suggests that a rapid increase in blood pressure overcomes the auto-regulatory mechanism of the cerebral vessels, causing cerebral hyperperfusion and damage to the capillary bed and then leakage of fluid into the interstitium. In the second theory, vasogenic edema is generated by endothelial cell activation, with subsequent cerebral vasoconstriction and hypoperfusion: cytotoxic chemotherapeutic agents and/or immunosuppressive therapy, infections and autoimmune diseases could induce endothelial dysfunction and subsequent cerebral edema. Concerning the field of this review, many potential triggers and risk factors for PRES such as hypertension, multidrug chemotherapy, HSCT, immunosuppressants, and graft-versus-host disease (GvHD) have been described. PRES may complicate chemotherapy treatment at any stage but the risk is increased during more intensive regimens (5). Different chemotherapeutic agents commonly used in children with hemato-oncological disorders are suggested to have toxic effect on endothelium. Recently, several cases of PRES have been described in children treated with molecularly targeted therapy as well (6, 7). In the setting of HSCT, PRES occurs usually within 100 days from transplantation; the use of calcineurin inhibitors for GvHD such as cyclosporine A or tacrolimus has been recognized as a major trigger for PRES. Acute GvHD, hypomagnesemia, the administration of fludarabine during conditioning and the use of umbilical cord blood stem cells are other factors associated with an increased risk of PRES in transplanted children (8).

Clinical features of PRES in children have been delineated in several studies. Clinical manifestations’ severity varies widely among patients; intensive care management may be required in selected cases to support vital functions (3). Symptoms of PRES may be preceded by tiredness and headache, usually reach their peak in during the first 48 h and improve during the first week. Complete clinical recovery is frequently achieved earlier than imaging resolution. Seizures are the most common, and often the presenting, manifestation of PRES (8). They typically manifest as non-convulsive seizures with occipital onset characterized by focal signs, such as gaze deviation, rhythmic ocular movements, and visual symptoms as hallucinations often associated with altered mental status of variable degree. Secondary generalization of seizures is common. Moreover, status epilepticus (SE), often with non-convulsive features, has been reported in these patients (9). Considering the difficulty in diagnosing non-convulsive seizures, EEG is useful in patients with PRES to detect subtle electrographic seizures and distinguishing between an epileptic and non-epileptic nature of specific neurological signs. Visual disturbances (e.g., cortical blindness, hemianopsia, and blurred vision) mental status changes until coma and nausea/vomiting and headache are other common symptoms of PRES. Rarely, children with PRES may show hemiparesis or other focal neurological signs (10).

**Figure 1:**
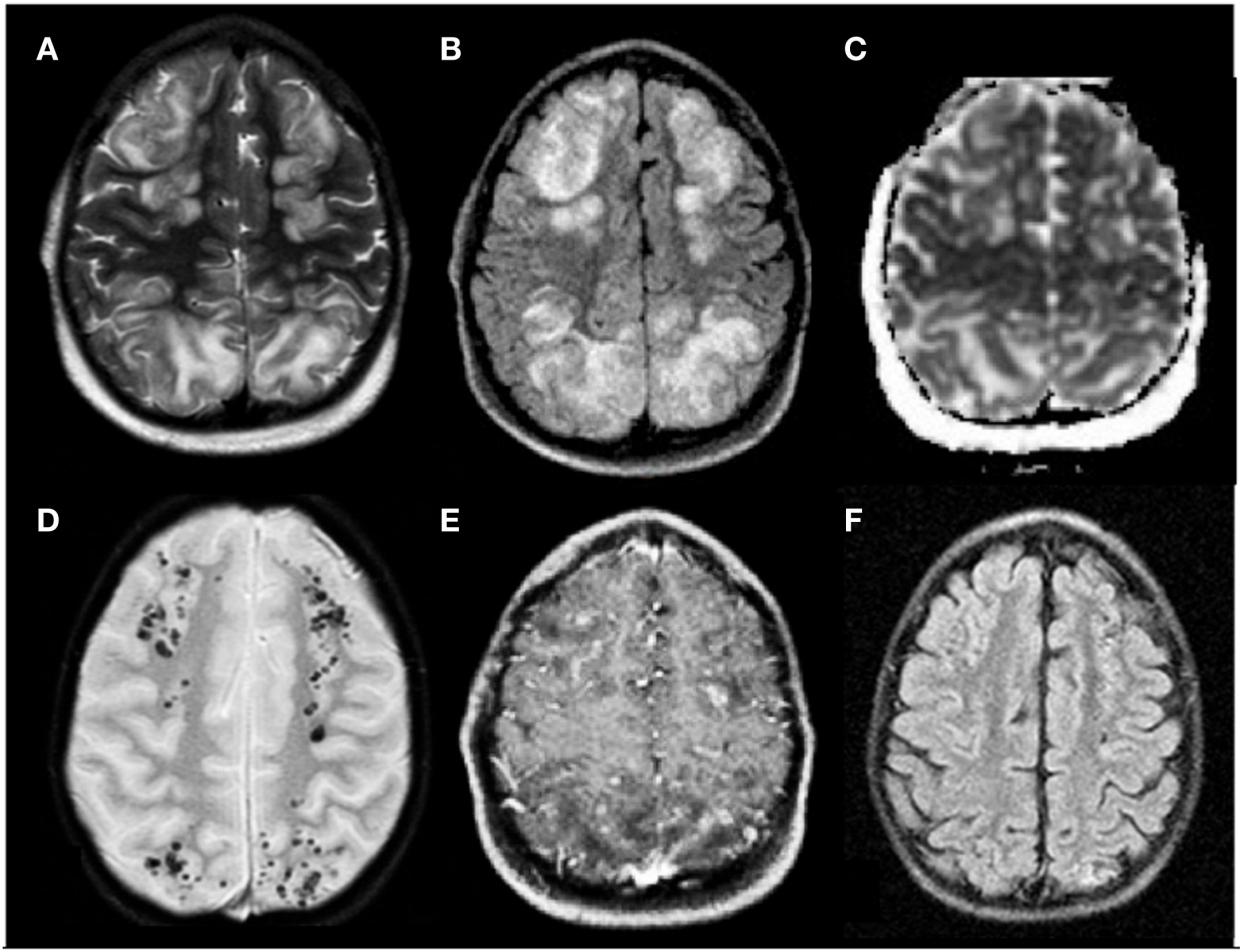
**Posterior reversible encephalopathy syndrome in a 5-year-old boy affected by neuroblastoma in treatment with vincristine, etoposide, and carboplatine**. **(A,B)** Axial T2 and FLAIR T2 images, respectively, display iuxtacortical hyperintensities that involve, bilaterally, frontal and parietal parasagittal watershed regions. **(C)** Diffusion study confirms they represent vasogenic edema. **(D,E)** Axial T2* and axial T1 post-gadolinium sequences, respectively, reveal microbleeds and faint signs of blood–barrier disruption within the cortex of affected regions. **(F)** Axial FLAIR T2 image acquired 1 month later shows complete resolution of the vasogenic edema.

As children receiving chemotherapy or HSCT can present with a wide spectrum of acute CNS complications, neuroimaging is a fundamental tool to correctly diagnose these events. Computed tomography (CT) is frequently the first study performed, but CT findings in PRES are sometimes normal or non-specific. Magnetic resonance imaging (MRI) is considered the gold standard for the diagnosis of PRES. In children with PRES, MRI typically shows high signal on T2-weighted images and FLAIR sequences, consistent with vasogenic edema involving mainly the subcortical white matter and frequently the cortex (11), as depicted in Figure 1. Diffusion-weighted imaging (DWI) is mandatory to differentiate PRES from cerebrovascular events, namely, ischemic stroke. The administration of gadolinium chelates can be required to exclude potential differential diagnosis [progressive multifocal leukoencephalopathy (PML), opportunistic infections]; in the majority of cases of PRES, gadolinium does not disclose any contrast enhancement but in some patients it may reveal mild signs of blood–brain barrier disruption. MRI shows in most patients a bilateral, sometimes asymmetrical, involvement of cerebral hemispheres. A predominant involvement of posterior regions of the brain (parietal and occipital lobes) is observed in most cases, while the frontal and temporal lobes are affected in approximately 50% of patients (12). In about one-third of cases, PRES involves the cerebellum, basal ganglia, and brainstem. PRES is considered to be a self-limited and benign entity; nevertheless, life-threatening events such as massive cerebral hemorrhages, cerebellar herniation, and refractory SE have been described as possible complications in pediatric patients diagnosed with PRES (13). Cerebral hemorrhage is reported accompanying PRES in 5–19% of cases (14, 15) and may present as small size hemorrhages (<5 mm), parenchymal hematoma, or subarachnoid hemorrhage. Intracerebral hemorrhages (ICHs) related to PRES are usually small, but massive, lethal ICHs have been reported. Notably, the risk of ICH is higher in children receiving allo-HSCT (16). Cerebellar herniation is a rare but catastrophic complication of PRES; it may happen as a result of severe edema of the posterior fossa structures. A rapid diagnosis of this life-threatening event is fundamental because of the possible need of surgical posterior fossa decompression. Outcome of PRES is usually good, and the reversibility of PRES has been widely described (8, 17); however, non-reversible cases in children with hemato-oncological disorders have been described, with the presence of long-lasting neurological sequelae or epilepsy secondary to residual brain lesions (17). Supportive care is the cornerstone of treatment. An intensive care unit admission can be required in selected cases. Antiepileptic drugs, in particular benzodiazepines, should be promptly administered to control ongoing seizures. If severe hypertension is found during the acute phase of PRES, a gradual decrease of the blood pressure would be advisable. Moreover, all patients should be evaluated for bleeding diathesis and for metabolic disturbances, in particular hypomagnesemia, either of which can require rapid correction. Finally, in most studies, withdrawal of treatments suspected to have caused PRES often resulted in the improvement of toxicity (18, 19).

## Therapy-Related Central Neurotoxicity Other than Pres

Many traditional and targeted therapies for hematologic and solid malignancies are associated with both peripheral and CNS toxicity. Peripheral neuropathy mainly associated with the use of vinca alkaloids is frequent during treatment for childhood cancer but is deliberately not addressed in this review. In the following pages, we will review CNS toxicity other than PRES associated with the most common chemotherapeutic agents. Main features are summarized in Table 1.

**Table 1:** **Toxicity associated with the most common chemotherapeutic agents used in pediatric onco-hematology**.

Neurologic toxicity	Neuroradiologic features	Risk factors and route of administration	Time of onset and duration	Incidence	Reference
**Methotrexate**					

Acute chemical meningitis	Thickened and gadolinium-enhancing nerve sleeves in case of adhesive arachnoiditis	Intrathecal (i.t.)	Onset within few hours with complete recovery in 2–3 days	5–40%	(20, 21)
Transverse myelopathy	Signal hyperintensity of the lateral and dorsal columns in T2-weighted magnetic resonance imaging (MRI), often with contrast enhancement (vacuolar demyelination and necrosis of the spinal cord)	i.t. often associated with i.t. cytarabine in heavily treated patients	Onset within hours or days with only some degree of recovery	Rare	(21–25)
(Sub)acute toxicity with stroke-like symptoms or seizure	Transient restricted diffusion on diffusion-weighted MRI, compatible with cytotoxic edema	i.t. or intravenous (i.v.) (moderate–high doses)	Brief episodes of symptoms few days/weeks after 2–3 courses	3–15%	(26–30)
Subacute leukoencephalopathy (LE)	White matter hyperintensity on T2-weighted and FLAIR MRI	Multiple courses of i.t. and i.v.	Development with repeated courses with variable persistence after the end of therapy	3.8% (symptomatic)–20% (asymptomatic)	(31–33)
Chronic LE	Periventricular white matter hyperintensity with possible temporary focal enhancement, ventriculomegaly and cortical atrophy	Repeated doses of i.t. or i.v. (high doses) but most frequent in combination and/or with brain radiotherapy	Onset several months to years after administration with variable clinical course	2% [i.v. methotrexate (MTX) alone]–45% (MTX + radiotherapy or i.t. MTX)	(20, 34, 35)

**Fludarabine**					

Delayed progressive LE (often with visual deficits)	MRI in T2 and FLAIR sequences shows mildly hyperintense lesions in the periventricular cerebral white matter, with restricted diffusion but no enhancement. Lesions are relatively mild comparing to clinical features.	High doses of i.v.	Delayed onset (21–60 days after treatment), progressive degenerative clinical course with short survival (median 2.2 months)	<1% with low doses (25 mg/m^2^/day × 5 days)–36% with high doses (>96 mg/m^2^/day × 5 days)	(36, 37)
Progressive multifocal leukoencephalopathy (PML) (JC V related)	MRI shows multiple patchy T2 hyperintense abnormalities of subcortical white matter, with no enhancement and no restricted diffusion	Standard doses i.v. or oral	Onset after 2 to several cycles of therapy with rapid and fatal clinical course (weeks to months)	Rare	(38, 39)

**Cytarabine**					

Acute reversible cerebellar syndrome	Normal computed tomography (CT) and MRI images	High doses i.v.	Onset of symptoms 6–8 days after initiating therapy, with resolution within 2 weeks	8–20%	(40, 41)
Myelitis/cauda equine syndrome	Possible spinal cord signal abnormalities with contrast enhancement on MRI images but often MRI images are normal	i.t. liposomal Ara-C (triple i.t. therapy or in combination with systemic high doses of MTX/Ara-C)	Delayed onset (1–91 days) after variable number (1–6) of i.t. courses. Incomplete recovery with severe and permanent sequela	10–28.5%	(23, 42–44)
Acute chemical meningitis	Thickened and gadolinium-enhancing nerve sleeves in case of adhesive arachnoiditis	i.t. liposomal Ara-C (higher risk without profilactic dexamethasone)	Transient episodes shortly after administration	~20% (with profilactic dexamethasone)	(23, 43, 45, 46)

**Blinatumomab**					

Acute reversible encephalopathy/seizure/cerebellar dysfunction	NA	Continuous i.v. infusion	Mostly mild episodes during the first cycle with complete resolution in few days. Usually managed without discontinuation of therapy	52% (any grade)	(47–49)
				13% (grade 3 or 4)	

**Brentuximab vedotin and rituximab**					

PML (JC V related)	MRI shows multiple patchy T2/FLAIR and diffusion-weighted imaging hyperintense abnormalities of subcortical white matter, with no enhancement	i.v.	Shorter latency for brentuximab (onset days-weeks after administration) compared to rituximab (median of 16 months). Rapid progression do death in a couple of months	Rare	(50–55)

**Ifosfamide**					

Ifosfamide-induced encephalopathy	Normal CT and MRI images	Oral or short time i.v. infusion	Acute reversible episodes starting during or few hours after administration and resolving in 20–72 h	10–40%	(56–59)

**Platinating agents (cisplatin, carboplatin, and oxaliplatin)**					

Peripheral sensory neuropathy and ototoxicity	Normal CT and MRI images	Dose-dependent, typically with cumulative cisplatin doses >400 mg/m^2^ (>60 mg/m^2^ for ototoxicity)	Deficits start during treatment and slowly progress in several months. Recovery is incomplete	47% (cisplatin)	(60, 61)
				97% (oxaliplatin)	

**Busulfan**					

Generalized seizure	Normal CT and MRI images	Dose dependent, usually associated with oral or i.v. high doses (>16 mg/kg)	Episodes are observed during the course or shortly after. Recovery is complete with no sequela	0.75–7.5%	(62, 63)

**Interleukin 2**					

Acute reversible encephalopathy	Possible transient T2 hyperintensities on MRI	IL-2 or PEG IL-2	2–22 days after starting treatment with gradual resolution in a few weeks	30–50%	(64, 65)

**Anti-GD2 monoclonal antibody (mAb)**					

Sensory–motor polineuropathy, hypertensive encephalopathy, ocular/visual abnormalities, inflammatory disease of central nervous system (CNS)	T2 hyperintensities with enhancement on MRI in case of inflammatory disease of CNS	Continuous i.v. infusion	Reversible toxicity during the infusion or shortly after	11–51% (ocular/visual abnormalities)	(66–68)

**Various agents: methotrexate, cytarabine, fludarabine, mAbs, cyclosporine, tacrolimus, rapamycine, sirolimus, azathioprine, etc**.					

Posterior reversible encephalopathy syndrome	Transient cortical/subcortical T2 hyperintensities on MRI, vasogenic edema	The risk is increased during more intensive regimens	Chemotherapy: at any stage	Chemotherapy: variable	(8)
			After hematopoietic stem cell transplantation (HSCT): days 0–100		
				After HSCT: 1–10%	
			Resolution: in 7–30 days		

**Asparaginase**					

Cerebral sinus venous thrombosis	Evidence of venous thrombosis on angio-MRI images	Acute lymphoblastic leukemia	During induction	About 3%	(69)

### Methotrexate

Methotrexate (MTX) is a folate analog, which inhibits the enzyme dihydrofolate reductase, blocking the *de novo* production of purines and thymidine. Moreover, it blocks the conversion of homocysteine to methionine and S-adenosyl-methionine, an important pathway in CNS myelination. Overall, it disrupts the important role of folate in synthesis and repair of DNA and CNS myelination. This drug is highly lipophobic; thus, it reaches the CNS only when given at high intravenous (i.v.) doses (>1 g/m^2^) or directly into the subarachnoid space. Intrathecal (i.t.) administration is mainly used in treatment or with prophylactic purpose of leptomeningeal spread of acute leukemia (AL), mainly acute lymphoblastic leukemia (ALL), or lymphoma. Several polymorphisms involving MTX transport and metabolism have been investigated as potential markers of heterogeneous toxicity and response to MTX. The non-synonymous C677T and A1298C variants in the 5,10-methylenetetrahydrofolate reductase are among the most widely studied. However, results are conflicting, main reasons being small and heterogeneous populations and differences in protocols and criteria defining toxicity (70, 71). Prevention of MTX neurotoxicity based on leucovorin rescue has been adopted in most protocols; however, its use is limited by rescue effect exerted even on leukemic cells and its efficacy in preventing neurotoxicity is partial (20).

Methotrexate neurotoxicity may be divided into acute, subacute, and delayed forms, either transient or chronic. i.t. administration is associated with the development of acute chemical meningitis in about 5–40% of patients, usually starting few hours after treatment and lasting up to 3 days. This usually consists of headache, stiff neck, fever, nausea, vomiting, and lethargy, generally self-limited. It is more common with no concomitant cranial irradiation. Adhesive arachnoiditis is the most severe form, which results in scarred tissue compressing nerve roots and their blood supply (20, 21).

**Figure 2:**
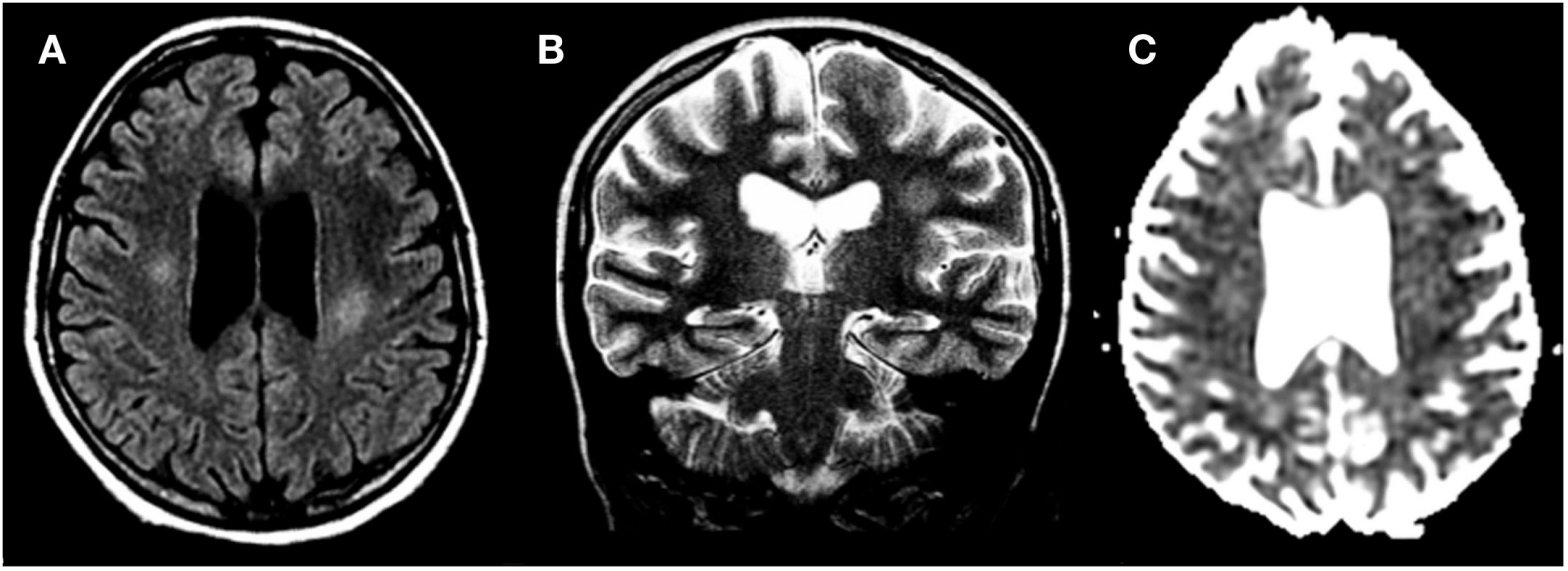
**Methotrexate-induced leukoencephalopathy in a 13-year-old girl with acute lymphoblastic leukemia**. **(A,B)** Axial FLAIR T2 and coronal T2-weighted images, respectively, reveal focal areas of hyperintensities within the deep cerebral white matter. **(C)** Diffusion study shows no cytotoxic edema.

Acute–subacute encephalopathy typically arises within few days to few weeks after i.t. or i.v. MTX administration. It consists of stroke-like episodes with transient neurologic symptoms such as hemiparesis, speech impairment, dysphagia, diplopia, hemisensory deficits, and sometimes seizure followed by complete recovery in a few days. Patients may be successfully rechallenged, but toxicity may recur. It is thought to be related to acute neuronal swelling caused by excessive stimulation of NMDA receptors by the high levels of homocysteine in cerebrospinal fluid (CSF). Dextromethorphan is a non-competitive antagonist of NMDA receptors and appears to be a promising agent in ameliorating symptoms and fastening recovery. DWI appears to be the most sensitive technique, revealing transient restricted diffusion, compatible with cytotoxic edema (27–31). In some patients, after repeated courses of MTX, radiologic evidence of leukoencephalopathy (LE) may be present, with white matter hyperintensity on T2-weighted and FLAIR MRI, as shown in Figure 2. of note, LE during active therapy also develops in about 20% of asymptomatic patients. These findings often persist at the end of therapy (22, 32, 33). Transverse myelopathy is another subacute complication arising few days to several months after i.t. MTX. Back or leg pain followed by paraplegia and sensory loss, flaccid paresis, and fecal and urinary incontinence/retention are typical features. This seems related to a non-inflammatory vacuolar demyelination and necrosis of the spinal cord, starting from the surface and progressing centrally. Demyelination is most prominent in the posterior funiculus, but it can also involve both the lateral and anterior funiculi. Elevation of protein level in CSF also occurs. T2-weighted MRI shows signal hyperintensity of the lateral and dorsal columns, with enhancement when contrast is used. Clinical course is often rapidly progressive, and recovery is often only partial (24, 25).

Methotrexate chronic LE is the major delayed complication following repeated courses of i.t. or i.v. MTX, but most commonly occurs with the combination of the two and radiotherapy. It may develop after months or even years from treatment. White matter damage leads to changes in mental status usually with preserved language. Main features are progressive cognitive impairment, loss of memory and concentration, changes in personality, dementia, seizures, paresis, and incontinence. Clinical course is variable but may be severe, also leading to coma and death. Neuroimaging shows white matter hyperintensity with possible temporary focal enhancement, usually involving periventricular areas. Ventriculomegaly with concomitant cortical atrophy is also seen (34, 35).

### Fludarabine

Fludarabine is a purine analog, mainly used in ALs and non-Hondgkin lymphoma, and in reduced intensity conditioning regimens prior to HSCT. Fludarabine-induced LE is peculiar because of its delayed onset (21–60 days from exposure), which can lead to difficulties in diagnosis. Pathophysiology is unclear but is probably due to direct cytotoxicity on axons and oligodendrocytes. Neurotoxicity appears to be strictly dose related, varying from very high incidence (36%) with high doses (>96 mg/m^2^/day × 5 days) to <1% with standard low doses (25 mg/m^2^/day × 5 days). Association of low doses of fludarabine with cytarabine is linked to a higher risk (3.6%) of neurotoxicity, particularly peripheral neuropathy. Clinical manifestations of LE include peripheral neuropathy, motor weakness, paralysis, pyramidal tract dysfunction, altered mental status, hallucinations, seizure, or extremely severe forms with several deficiencies worsening progressively to coma and death in a few weeks or months. Clinical course is characterized by progressive degeneration with short survival (median of 2.2 months). Resolution is rare and, when occurs, several dysfunctions are usually left over. No prophylaxis or treatment is known to date. A classic feature is visual disturbances resulting from cortical blindness, visual pathway demyelination, and/or retinal damage. Pathology reveals axonal and myelin damage in the periventricular matter of the brain and spinal cord, with vacuolization and macrophage infiltration. MRI abnormalities are relatively mild compared with clinical severity and usually do not show spinal cord involvement. T2 and FLAIR sequences show mildly hyperintense lesions in the periventricular cerebral white matter, with restricted diffusion but no enhancement. Sequential MRI images reveal increasing size and intensity of the lesions (36, 37). PML caused by JC virus after standard-dose fludarabine is another possible complication. Only few cases have been described. Lymphopenia lasting up to a year after therapy increases the risk for opportunistic infections, which seem a plausible cause for PML. The resulting syndrome is similar to other causes of LE (see Brentuximab Vedotin and Rituximab), with a rapid worsening course conducting to death. Up to date, no treatment is available (38, 39).

### Cytarabine

Cytarabine (Ara-C) is a nucleotide analog frequently used in the treatment of leukemia and lymphoma, especially in case of CNS involvement and brain tumors. When given i.v., it is fast eliminated by hepatic cytidine deaminase, but this enzyme is not present in the CNS, so cytotoxic levels in CNS persist longer when given i.t. or i.v. at high doses (>3 g/m^2^). Liposomal Ara-C is a slow release formulation, which allows maintenance of therapeutically effective concentrations up to 40 times longer than standard Ara-C, with a better response rate. However, it has to be administered in association with corticosteroid in order to reduce the development of arachnoiditis. Neurological side effects of i.t. liposomal Ara-C are similar to that of i.t. MTX. Despite prophylactic treatment with dexamethasone, chemical meningitis with nausea, fever, vomiting, and back pain may develop in 20% of patients. More severe complications are spinal cord lesions (cauda equina syndrome, paraplegia, or tetraplegia) and papilledema, occurring at a median of 10 days (1–22, 24–59, 62, 63, 70–100) after a variable number of courses. For these, recovery is often incomplete with severe neurologic deficits after many months and no effective therapy available. Risk is particularly high when i.t. liposomal Ara-C is administered in association with i.v. high dose of MTX and/or Ara-C or in combination with i.t. MTX and steroids (“triple therapy”). MRI may show contrast enhancement of lateral columns and cauda equina but may also be non-informative. Finally, altered mental status and seizure are further possible complication of i.t. liposomal cytarabine (42–46, 72). Acute cerebellar toxicity is a typical complication of high dose i.v. cytarabine due to direct damage of Purkinje cells. Cerebellar signs such as nystagmus, ataxia, dysarthria, and oculomotor impairment, rarely with confusion and drowsiness (40). Symptoms are usually mild and reversible with in two weeks of discontinuing the drug but in some cases, permanent impairment has been described. CT and MRI images are usually normal. The most important risk factors, besides cumulative dose (>36 g/m^2^), are age (>50 years) and renal dysfunction (41).

### Blinatumomab

Blinatumomab is a bispecific T-cell-engaging antibody which allows the interaction between normal CD3+ T cells and CD19+ acute lymphocytic leukemia cells, leading to a lysis of the tumor cells. It is usually administered as continuous i.v. infusion over 4 weeks. Clinical trials have proven significant response rate in refractory or relapsed B-ALL. Neurotoxicity is rather common, reaching 52% (13% of grade 3 or 4 neurologic events), but it is usually mild and reversible. Moreover, usually it can be managed with dexamethasone treatment without infusion discontinuation. CNS toxicity may consist of headache, somnolence, confusion, aphasia, syncope, seizure, or cerebellar symptoms such as ataxia or tremor with a median time of onset of 7 days. The underlying mechanisms remain unclear (47–49, 73).

### Brentuximab Vedotin and Rituximab

Progressive multifocal leukoencephalopathy is a rare, often fatal, demyelinating disease of the CNS resulting from infection of oligodendrocytes by JC polyoma virus (JCV). This is a complication associated with immunosuppressive condition, such as lymphoproliferative disorders, autoimmune diseases, human immunodeficiency virus infection or, as described in the last decade, treatment with monoclonal antibodies (mAbs) such as brentuximab vedotin and rituximab. Brentuximab vedotin is a CD30-specific antibody drug conjugated with the microtubule inhibitor monomethyl auristatin E employed in treatment of relapsed-refractory Hodgkin lymphoma and anaplastic large cell lymphoma. Rituximab is a chimeric anti-CD20 mAb approved for CD20-positive B cell non-Hodgkin’s lymphoma and chronic lymphocytic leukemia. Pathogenesis of PML is uncertain, probably related to reactivation of the virus in a setting of altered immune surveillance: while brentuximab causes CD30+ activated T-cells depletion, rituximab does not result in T-lymphocyte depletion and PML may result from expansion of pre-B-lymphocytes harboring latent JC virus after rituximab mediated B-lymphocyte depletion (50, 51). Clinical presentations include aphasia, hemiparesis, hemianopsia, memory loss, gait dysfunction, and confusion with a rapid and progressive worsening leading to death over a period of a couple of months. Time of onset is relatively short in brentuximab-related PML (days to weeks after the administration of two to six doses) compared to rituximab-related PML (median of 16 months after median of six doses). Case fatality rate is extremely high (80% for brentuximab, 90% for rituximab), with death in few months from presentation (52–55). Many foci of demyelination are seen as increasingly confluent white matter abnormalities on MRI, especially in the subcortical areas of cerebral hemispheres. JCV DNA is often not detectable in CSF. There is no standardized approach to PML. Discontinuation of the immunosuppressive agent or cytarabine is often adopted, although benefit is rarely observed. An immune reconstitution inflammatory syndrome characterized by rapid infiltrates of T-cells may develop. This can not only be life saving, due to the reconstitution of the immune system, but also lethal for excessive inflammation (55).

### Ifosfamide

Ifosfamide is an alkylating agent mainly used in treatment of relapsed ALL, lymphoma, sarcoma, and other solid tumors. CNS toxicity is frequently observed (10–40%) with high dose treatment. It happens more frequently after oral than after i.v. administration, or, when given i.v., it occurs more frequently with a short infusion time. Risk factors to develop ifosfamide-induced encephalopathy (IIE) appear to be related to altered renal function, hepatic metabolism, and, as recently reported, to specific ifosfamide formulation (74). The more widely accepted explanation is toxicity of one or more of the ifosfamide metabolites, particularly chloroacetaldehyde. This crosses the blood–brain barrier and exerts direct neurotoxic effect, depletes CNS glutathione, and inhibits mitochondrial oxidative phosphorylation. IIE ranges from mild confusion (the most frequent symptom) or somnolence to more severe forms with memory loss, disorientation, hallucinations, seizure, delirium, or, rarely, even coma and death. Cerebellar and cranial nerve dysfunction or other movement disorders have rarely been described (56, 57). Symptoms occur during infusion or after few hours, followed by complete recovery in 20–72 h. Additional treatment generally is not necessary but i.v. methylene blue, 50 mg every 4–8 h, has been used. However, it is unclear if clinical symptoms resolved independently of treatment. There seems to be little advantage in using methylene blue during subsequent infusions of ifosfamide, mainly consisting of shorter duration and lower grade of toxicity but not in reducing the risk of recurrence. CT and MRI imaging, when performed, do not reveal any abnormalities (58, 59).

### Busulfan

Busulfan is an alkylating agent, largely used in conditioning regimens prior to HSCT. High doses (>16 mg/kg) are a good alternative to total body irradiation in myeloablating regimens, while lower doses (3.2–6.4 mg/kg) are used in combination with melphalan and fludarabine in reduced intensity conditioning regimes. Neurotoxicity is a well-known side effect, mainly consisting of generalized seizure in the third or fourth day of administration without any sequela. This complication appeared to be dose dependent and related to the high CSF concentrations of this small, lipophilic molecule during systemic administration. Anticonvulsant prophylaxis is now considered the standard of care in pediatrics, although the real utility is not fully understood (62, 75).

## Infectious Complications

Infections of CNS occur in a relevant proportion of pediatric onco-hematological patients and contribute significantly to morbidity and mortality. The incidence of CNS infection evaluated in the more widely studied adult population ranges from 0.8 to 15% (63).

Central nervous system infections are more common in patients with hematological malignancies undergoing HSCT, albeit less frequent, infections in patients with solid tumors or non-malignant hematological disorders are possible (76). Pediatric patients with cancer, and especially those with hematological disorders, are frequently severely immunocompromised with defects in anatomical and functional (humoral and cellular) elements of immune response. These defects might either be due to the hematological disorder itself or secondary to the antineoplastic treatment such as neutropenia, effects from irradiation, systemic immunosuppression, or HSCT (77). Moreover, extraordinary supportive cares keep patients alive for longer periods of time offering a wider possibility for possible colonization with multiresistant or unusual pathogens. The agents that cause such infections are often opportunistic organisms, and various causative pathogens, including bacteria, fungi, viruses, and protozoa, have been identified (78). Since neurological symptoms could be non-specific CNS infection justify a high degree of awareness. In immunocompetent hosts, CNS infections are broadly classified into meningitis, meningoencephalitis, and cerebritis/abscess formation; on the other hand, reduced inflammatory reaction in immunocompromised patients can induce only faint symptoms (79). In addition, pathogens determine infections in specific patient groups based on the pattern of immunosuppression (deficiency in cell-mediated immunity vs inadequate humoral immunity) (80). Neutropenic patients generally experience bacterial, fungal, and viral CNS infections. Lack of T cell immunity or in macrophages activity predispose to cerebral toxoplasmosis (80). Adequate diagnostic techniques including neuroimaging, CSF examination, and, in carefully chosen cases, biopsy of focal mass should immediately be achieved in case of any suspicion of CNS infection. CSF analyses including staining and microscopy, culturing, serological techniques, and polymerase chain reaction (PCR) assays are mandatory to recognize meningoencephalitis, which is typically caused by viruses, *Candida* spp., and bacteria. Focal lesions, typically caused by *Toxoplasma* or *Aspergillus* spp. could require brain biopsy. Routine parameters in the CSF are frequently non-specifically altered in these patients. Neuroimaging should commonly rely on MRI since it is more sensitive than CT scan for diagnosis of the majority of CNS infections (81, 82). On the other hand, despite the ionizing radiation issue, CT is faster to achieve and most widely widespread, thus becoming an important tool in the acute setting. Severe CNS bacterial infections (i.e., caused by *Neisseria meningitidis* and *Streptococcus pneumoniae*) can be present at the diagnosis of ALs but usually are not dissimilar from those commonly described. For this reason, in the next sections, we will focus on the relevant features of fungal, viral, and parasitic CNS infection most frequently encountered in pediatric patients undergoing chemotherapy and/or HSCT.

### Fungal Infection

The predominant fungal pathogen is *Aspergillus* spp., with *Aspergillus fumigatus* prevailing over other species. *Candida* spp., and Mucorales, may less frequently be encountered (83). In the past decade, annual incidence of invasive aspergillosis was 0.4% of hospitalized immunocompromised children (84). Although CNS aspergillosis has traditionally been associated with a dismal prognosis, during the last 25 years, there has been a drastic mortality reduction from more than 80% to less than a half. This decrease may be explained by CNS aspergillosis earlier diagnosis as well as the development of amphotericin B lipid formulations and the introduction of azoles (85–87). Prognosis correlates primarily with immune status, with allo-HSCT patients having sixfold greater odds of death (88). Aspergillar fungal hyphae have a characteristic angiotropism: they form broad hyphae that invade medium to large arterial and vein vessels, leading to acute infarction and hemorrhage. Afterward they extend into surrounding tissue inducing an infectious cerebritis or progressing into an abscess. Rarely, CNS aspergillosis presents with overt meningitis or cause granuloma (89). The clinical picture is usually characterized by fever, seizures, mental status alteration, visual deficits, and focal neurologic signs (89).

**Figure 3:**
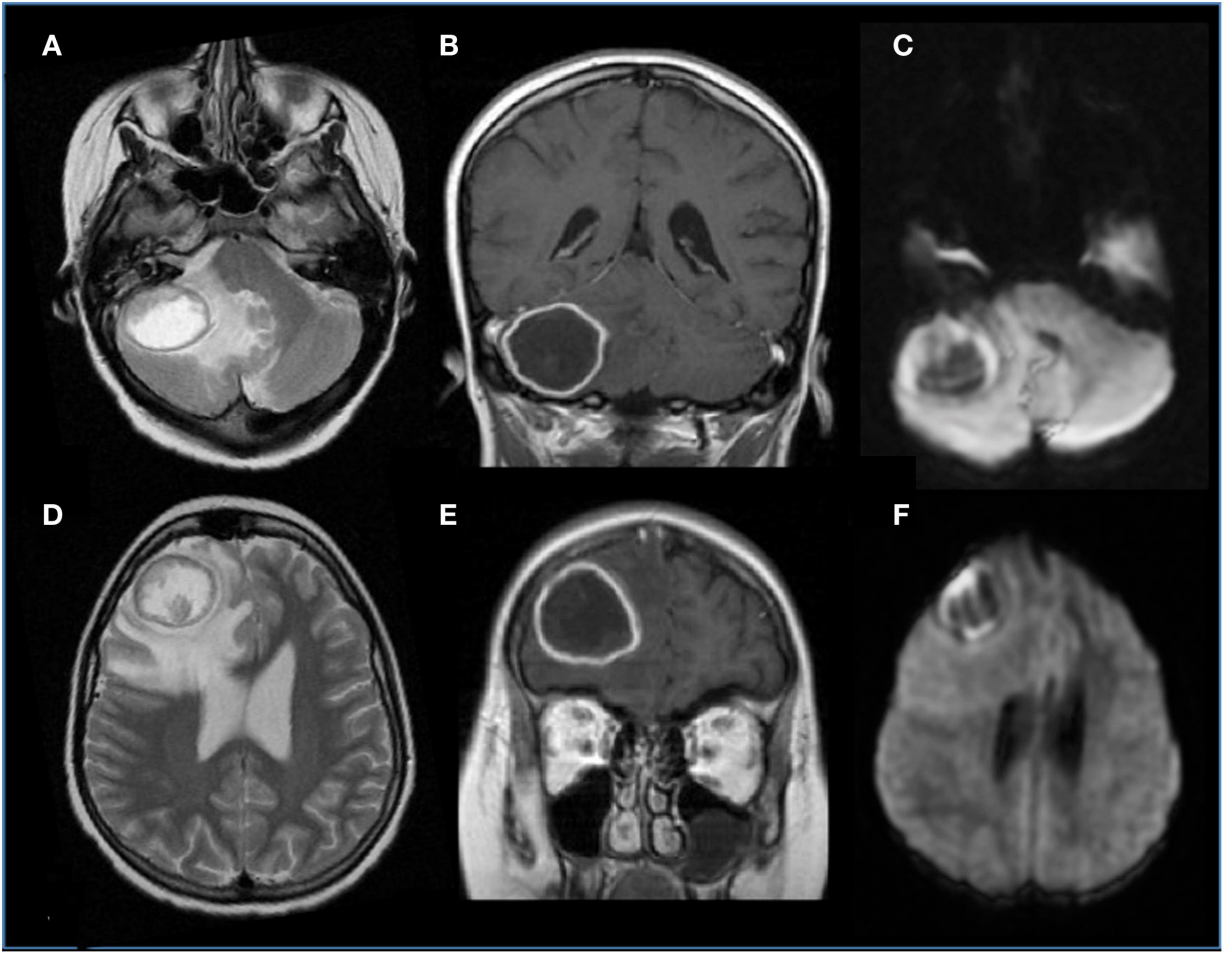
**Brain aspergillosis in a 15-year-old girl with acute lymphoblastic leukemia**. **(A,B,D,E)** Axial T2 and coronal post-gadolinium T1-weighted images, respectively, showed ring-enhancing lesions with mass effect, surrounded by abundant edema, on the right side, within the frontal lobe and cerebellar hemisphere. **(C,F)** Diffusion study reveals marginal hyperintensities representing peculiar intracavitary fungal hyphae projections.

Prompt diagnosis is important given the high morbidity and mortality associated with this infection, but many children with suspected invasive mold infections do not have an identified pathogen, and are treated empirically. Although culture from a sterile site remain the gold standard for diagnosis and provide antifungal susceptibilities information, invasive procedures are needed and the diagnostic yield is low. In addition, unfortunately *Aspergillus* is rarely recovered from blood cultures (90). Thus in many patients the diagnosis relies on indirect findings including fungal markers (galactomannan antigen and 1,3-β-d-glucan) and radiology. Typical neuroimaging features are shown in Figure 3 and include ring-enhancing lesions, with a central T2 hypointensity area, associated with hemorrhagic foci and peculiar intracavitary projection at the diffusion study, representing fungal hypae (91). The degree of immunocompetence of the host is tightly connected with the level of contrast enhancement of the lesion. As *Aspergillus* has a peculiar “philia” for perforating arteries, thalami, basal ganglia, and corpus callosum, as well as subcortical regions represent usual sites of involvement. Due to its angioinvasive nature, arterial infarction (lacunar and territorial), vasculitis, and mycotic aneurysm can be encountered (92). Candidiasis has a different appearance, vasculitis, hemorrhage, and thrombotic infarction are less commonly encountered, and it comprises multiple ring-enhancing micro-abscesses at the cortico/medullary junction, cerebellum, and basal ganglia (93).

### Viral Infection

Viral infection caused by herpesviridae (herpes simplex virus, Epstein–Barr virus, varicella zoster virus, cytomegalovirus—CMV, and HHV-6) and polyomaviridae (JC polyoma virus—JCV) families are more frequently encountered in allo-HSCT recipients (94). Viral PCR assays on CSF samples have high sensitivity and specificity, thus regarded as a gold standard for diagnosis (95).

HHV-6 primary infection may be asymptomatic or may cause roseola infantum (sixth disease), a febrile exanthema. The virus achieves latency in the salivary gland, white blood cells, and the brain. The clinical presentation of HHV-6 encephalitis is characterized by temporal lobe seizures, fever, and mental status changes that include insomnia. Diagnosis is reliably established by PCR analysis for HHV-6 in CSF, and immunohistochemistry is used to confirm the presence of HHV-6B protein antigens. Prompt therapy with ganciclovir or foscarnet significantly improves outcome (96). Neuroimaging studies may be normal in the early stage of symptomatic infection. The most commonly reported MRI pattern consists of generally confluent lesions, involving the mesial temporal lobes and limbic system, that are hyperintense on T2-weighted images. The lesions usually do not enhance in T1-weighted images (97). Unusual but devastating form of primary HHV-6 infection reported in infants is characterized by acute necrotizing encephalopathy with symmetric involvement of the basal ganglia, thalami, and brainstem (98). Diffusion restriction may affect the mesial temporal lobe and the basal ganglia (98).

Cytomegalovirus, also termed human herpesvirus-5, is one of the most ubiquitous members of the Herpesviridae family. An estimated 90% of the population is infected worldwide. Endogenous reactivation may occur in transplanted patients. CMV primary infection in a seronegative host after receiving a CMV-seropositive transfusion or HSCT represents the most adverse presentation (99, 100). The virus can affect both the CNS and the peripheral nervous system. Chorioretinitis and myelitis–radiculitis, followed by encephalitis and ventriculitis are the most common forms of neurologic involvement. The virus can affect both the CNS and the peripheral nervous system. Chorioretinitis and myelitis–radiculitis, followed by encephalitis and ventriculitis are the most common forms of neurologic involvement. CMV ventriculitis is characterized by thin, smooth ependymal enhancement with associated debris within the ventricles. Despite not specific, retinal involvement or polyradicular or ependymal enhancement should raise suspicion of CMV infection (101). Ganciclovir (and its orally available prodrug, valganciclovir) and foscarnet are the most effective first-line agent for CMV treatment after HSCT.

JCV is a neurotropic polyomavirus whose only known reservoir is humans. It is the causative agents of PML, a demyelinating disease of the CNS with focal or multifocal neurologic deficits due to virus reactivation (102). JCV seroprevalence rises from about 16% in children 1–5 years of age to 34% by age 21–50 years, which may explain why PML is relatively uncommon among children (103). PML has been discussed in more details in section dedicated to brentuximab vedotin and rituximab toxicity.

### Parasitic Infections

*Toxoplasma gondii* is an intracellular protozoan that is found worldwide and represents the pathogenetic agent of CNS Toxoplasmosis. The mortality rate is extremely high (60–90%), despite specific therapy (104). Clinical manifestations may span from fulminant, disseminated infection to disease confined to the brain. In patients receiving HSCT, the incidence of toxoplasmosis has been reported up to 6% (105). The vast majority of cases are reactivation of a latent infection in patients receiving allo-HSCT, while is rare after autologous HSCT. Cord blood transplantation may be a risk factor (106). MRI is characterized by ring-enhancing lesions—often multiple, but solitary in 30% of the cases—involving more commonly thalami and basal ganglia. An eccentric target sign has been described on post-contrast images, which presents high specificity (95%) and a rather low sensitivity (30%) (107). MR spectroscopy reveals elevated lipid/lactate peak. CNS lymphoma represents the main differential diagnosis in patients suspected of having CNS toxoplasmosis. CNS Lymphoma represents the main differential diagnosis in patients suspected of having CNS toxoplasmosis. Positron emission tomography with fluoro-deoxy-glucose commonly demonstrates absent glucose consumption in *Toxoplasma* lesion, characteristic of its non-malignant behavior, thus differentiating it from lymphoma (108). Pyrimethamine and sulfadiazine are usually given for treatment of cerebral toxoplasmosis and the occurrence can be significantly reduced by the use of trimethoprim–sulfamethoxazole prophylaxis.

## Cerebrovascular Complications

Hematologic malignancies show the strongest association with cerebrovascular complications followed by brain tumors (mass effect). The prevalence of stroke in children with cancer is approximately 1%, with essentially the same frequency for hemorrhagic and ischemic stroke. Stroke is estimated to happen at a median of 5 months after cancer diagnosis, though the range of time presentation was broad (109). Cerebrovascular events may be related to not only several factors, such as cancer itself, the presence of CNS metastasis, infectious events but also treatment directly or through vascular injury (110).

### Intracranial Hemorrhage (ICH)

**Figure 4:**
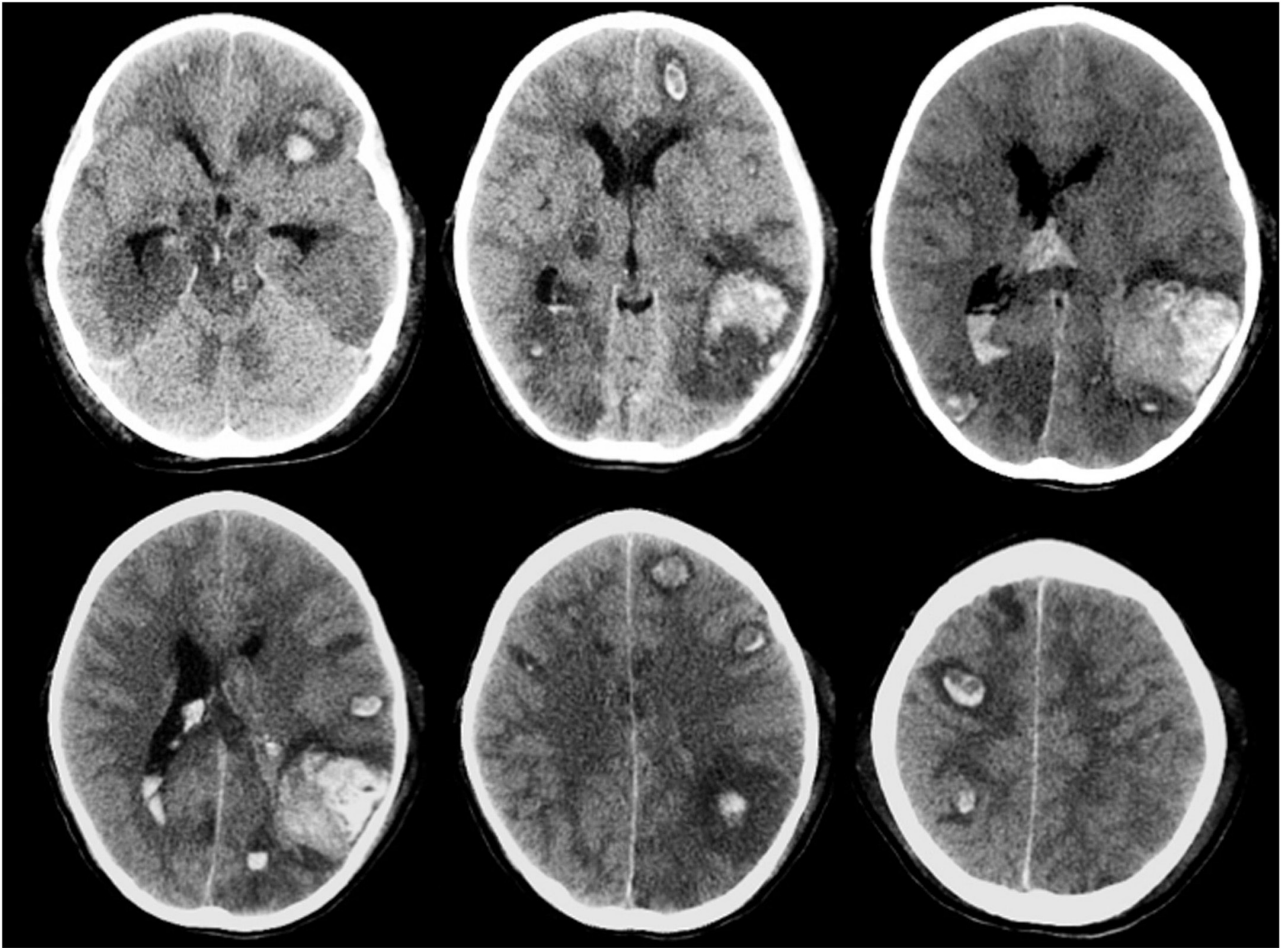
**Multiple brain hemorrhage in a 12-year-old girl affected by acute myeloid leukemia**. Axial computed tomography images show multiple hemorrhagic foci involving mainly the subcortical areas of the cerebral hemispheres, more evident in the left temporo-parieto-occipital region.

Intracranial hemorrhage is the most frequent and severe hemorrhagic complication in patients with AL, representing one of the main causes of mortality of these patients. The highest incidence is observed in acute myeloid leukemia, especially the promyelocytic subtype (111, 112). Often it occurs at the onset or during induction therapy as a consequence of the disease itself, generally associated with thrombocytopenia and coagulopathy. Among described risk factors are prolonged prothrombin time, female gender, thrombocytopenia, and above all hyperleukocytosis. Also, asparaginase (ASP) treatment has been associated with increasing risk of ICH in some reports (113). ICH is more often localized in the supratentorium but could also involve basal ganglion, cerebellum, and brainstem, often with multiple sites. Cortical hemorrhage is the most frequent with not only parietal involvement as typical site but also subarachnoid hemorrhage, subdural hemorrhage, and epidural hemorrhage can occur (113). Brainstem, subarachnoid, and epidural hemorrhages are strongly related to early death. A CT scan of a patient with multiple hemorrhagic foci is shown in Figure 4. Seizures, focal neurological deficits and impaired mental status are major clinical manifestations depending on cerebral area of stroke. Prompt CT scan at the onset of signs/symptoms could be very useful in the diagnosis and management of ICH, allowing a rapid neurosurgical approach if needed. In AL, controlled decrease in the circulating leukemic blast cells and correction of coagulopathy should be appropriate treatment to avoid this complication (110).

### Thromboembolic Events

**Figure 5:**
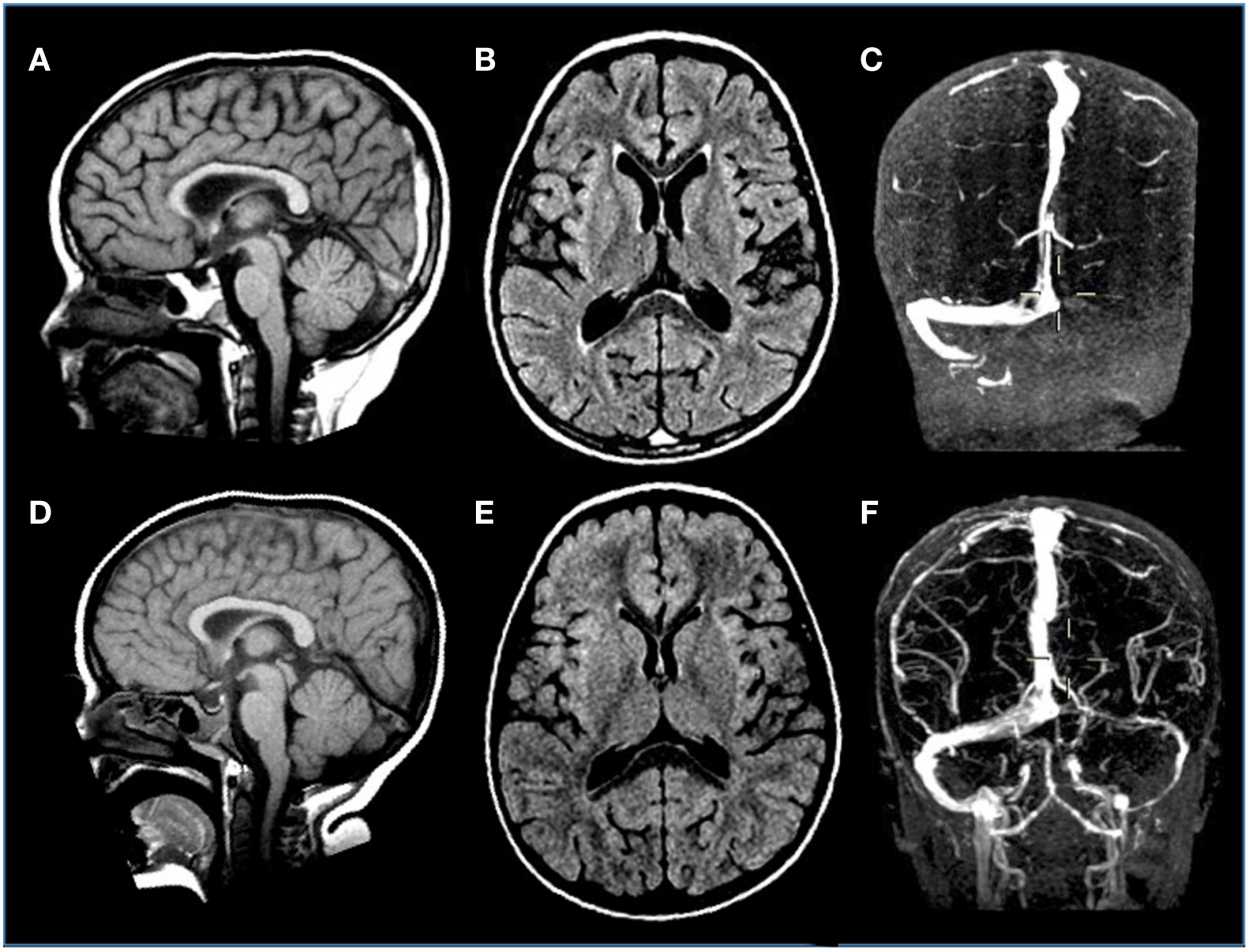
**Venous sinus thrombosis in a 4-year-old boy with acute lymphoblastic leukemia treated with l-asparaginase**. **(A–C)** Sagittal T1, axial FLAIR T2-weighted, and coronal angio-MR images display thrombosis of the distal portion of the superior sagittal and left transverse sinuses. **(D–F)** Sagittal T1, axial FLAIR T2-weighted, and coronal angio-MR images acquired 3 weeks later reveal resolution of the thrombosis with almost complete restoration of flow signal within the previously thrombosed sinuses.

Incidence of symptomatic deep venous thrombosis in pediatric oncology patients is described between 7 and 14%, while asymptomatic ones are estimated in over 40% of cases. Different risk factors are reported including age (<2 years or >10 years), non 0-blood group, presence of central venous cine, immobility, infections, obesity, underlying thrombophilia, corticosteroids, and ASP therapy. The thrombosis can occur both in central and peripheral veins, occasionally involving the arterial system. Cerebral sinus venous represents the main CNS localization (Figure 5), accounting for about 8% of all thrombotic events (114). As regard ALL, the Prophylactic Antithrombin Replacement in Kids with ALL treated with Asparaginase study reported a prevalence of asymptomatic thrombosis of 36.7% as opposed to only 5% of symptomatic events (115). Other studies confirm that incidence of symptomatic thrombotic events is about 5%, with CNS and upper extremity being the most more frequent districts involved (69, 116, 117). Clinical manifestations of cerebral sinus venous thrombosis is altered mental status, headache, vomiting, and diplopia. Seizures and other focal neurological deficits could also occur. In particular, the use of ASP in patients with ALL is associated with a risk of thrombosis. Usually, this complication occurs during induction phase. An imbalance of the pro- and anticoagulating systems with reduction in fibrinogen, V, VII, VIII, and IX factors but also antithrombin III (AT), protein S, protein C, plasminogen, and alpha-2-antiplasmin has been reported in patients treated with this drug (118). So far, although recent paper highlighted a lower thrombin generation profile with pegylated *Escherichia coli* asparaginase (PEG-ASP) than with native ASP, no difference has been demonstrated in influencing thrombotic complications in clinical trials between the different ASP formulations, as native *Escherichia coli*-ASP and PEG-ASP. Few data are available about adverse effects of Erwinia ASP, but no significant differences with E. Coli ASP profile were found (119–121). Concomitant use of corticosteroids and ASP has been investigated as a worsening factor for thrombotic events but no agreement was achieved (122).

Several strategies have been investigated to reduce the risk of developing thrombotic complications during treatment with ASP with controversial results. Prevention therapy with fresh frozen plasma or cryoprecipitate replacement was proposed without successful results (123). Prophylaxis using low molecular weight heparin (LMWH) was also examined with positive effects reported in a non-randomized study of 41 pediatric patients with ALL (124). However, additional study did not prove the same results (125, 126). Trials with preventive supplementation of AT have been trying with promising outcomes but consistent data are lacking (127). Prophylaxis with combination of LMWH and AT replacement also appears as a valid therapeutic strategy (128). As concern, management of thrombosis with LMWH is usually the first choice, even if unfractionated heparin is more appropriate in patients at a high risk of hemorrhage or with renal failure. Monitoring of AT levels and replacement when needed should be useful to maintain heparin anticoagulation. Efficacy and safety of new oral drugs such as rivaroxaban or dabigatran could be evaluated, although their irreversibility is a potential contraindication in this kind of patients.

## Author Contributions

All the authors listed have made substantial, direct, and intellectual contribution to the work and approved it for publication.

## Conflict of Interest Statement

The authors declare that the research was conducted in the absence of any commercial or financial relationships that could be construed as a potential conflict of interest.
